# Outcomes of Hypofractional Tomotherapy in Patients with Stage III Nonsmall Cell Lung Cancer Who Are Not Eligible for Surgery or Concurrent Chemoradiation

**DOI:** 10.1155/2020/9168424

**Published:** 2020-06-30

**Authors:** Jing Li, Hongqi Li, Yingjie Wang, Junyang Liu, Xuan Wang, Haifeng Pang, Dongshu Chang, Yupeng Di, Gang Ren, Ping Li, Yong Wang, Chen Liu, Xiao Chen, Xiaoli Kang, Tingyi Xia

**Affiliations:** ^1^Medical School of Chinese PLA, No. 28 Fuxing Road, Beijing 100853, China; ^2^Department of Radiation Oncology, Airforce Medical Center, PLA, No. 30 Fucheng Road, Beijing 100142, China

## Abstract

**Purpose:**

We assessed the clinical outcomes and toxicities following hypofractionation with helical tomographic intensity-modulated radiotherapy technology (tomotherapy) in patients with stage III non-small cell lung cancer (NSCLC) who were not candidates for surgery or concurrent chemoradiation.

**Methods:**

Forty-three patients with stage III NSCLC who were treated between 2011 and 2017 were enrolled. The prescription doses for gross target volume and clinical target volume were 70 Gy and 60 Gy (respectively) delivered in 15–25 fractions over 3–5 weeks.

**Results:**

The median overall survival (OS) time was 34.23 (range 11.33–99.33) months. The estimated 1-, 2-, and 3-year OS rates were 97.7%, 74.4%, and 55.9%, respectively; the corresponding progression-free survival (PFS) rates were 79.1%, 53.5%, and 36.1%, respectively. The local disease recurrence, regional disease recurrence, and distant metastasis rates at 3 years were 4.7%, 11.62%, and 55.81%, respectively. On multivariate analysis, dose regimen (<19 f vs. ≥19 f) was an independent prognostic factor affecting OS, PFS, and DM (*p* < 0.05). Seven patients developed grade 1-2 acute radiation pneumonia (RP), 5 patients developed grade 1-2 late RP, while 3 patients developed grade 3 late RP. None of the patients developed grade 4-5 radiation lung injury.

**Conclusion:**

Tomotherapy may be an effective treatment option for patients with stage III NSCLC. It may be a viable alternative to surgery with lower incidence of side effects.

## 1. Introduction

Currently, stereotactic radiotherapy is becoming a viable alternative to surgical treatment of early-stage non-small cell lung cancer (NSCLC). The clinical application of molecular targeted therapies and immunotherapy have significantly prolonged the survival time of patients with advanced-stage NSCLC. For most patients with locally advanced NSCLC, the current standard is chemoradiotherapy (conventional radiotherapy 60–66 Gy/30–33 f), which has not changed for more than 40 years [1]; the associated median survival time is only 19 months and the 5-year survival rate is 13–16% [2]. Even in patients with stage IIIa NSCLC who can be operated, the 5-year survival rate is only up to 25–30%, while the failure rate of local treatment is as high as 30–40% [3, 4]. Therefore, it can be said that the failure of local control implies the failure of the whole treatment. With the advances in modern radiotherapy technology, increased single dose and shorter treatment duration of radiotherapy can improve the tumor local control rate and overall survival time [5].

In a previous study conducted at our department, the 3-year survival rate of patients with stage I NSCLC was 91% [6]. Chang et al. showed that in patients with early nonoperable NSCLC, hypofractional radiotherapy (54 Gy/3 f or 50 Gy/4 f) can achieve better survival benefit and less toxic effect than surgery [7]. However, due to the large volume of tumor or the number of metastatic lymph nodes in patients with stage III NSCLC, there is limited scope for increasing single dose. Nowadays, with the significant advantage of 360 degrees rotation, use of unique binary pneumatic multileaf collimator, and 51 field beam angles, helical tomographic intensity-modulated radiotherapy technology (tomotherapy) can deliver higher dose for the target, while protecting the normal lung tissues; this can greatly improve the tumor conformability and dose homogeneity.

Therefore, the purpose of this study was to explore the feasibility and effectiveness of hypofractional tomotherapy in patients with stage III NSCLC who are not eligible for surgery or concurrent chemoradiation. The primary end points were overall survival (OS) rate and progression-free survival (PFS) rate. Local control rate, regional control rate, distant metastasis rate (DM), and incidence of radiation pneumonitis (RP) were regarded as secondary end points.

## 2. Materials and Methods

### 2.1. Patients and Study Design

A total of 43 patients with stage III NSCLC who were treated between December 2011 and March 2017 were enrolled in this study. The inclusion criteria were as follows: (1) patients with histologically or cytologically confirmed NSCLC who were inoperable or refused surgery; (2) Karnofsky performance score (KPS) >70; (3) patients who were able to lay on the bed for more than 30 minutes; (4) use of 18-Fludeoxyglucose positron emission tomography (18F-FDG PET)/CT and CT scans for stage III patients within 1 month before tomotherapy; (5) union for International Cancer Control (UICC) TNM classification system (2002) was used for staging; (6) patients who had previously received chemotherapy, except bleomycin, were also allowed in this study. Genetic testing was recommended for patients with adenocarcinoma and targeted therapy (such as gefitinib or erlotinib) was recommended as maintenance therapy in patients with EGFR-mutant or EML4-ALK fusion gene. Patients who received other treatment for NSCLC, those with history of any cancer in the 5-year period immediately preceding the confirmation of NSCLC, those with a history of chest radiotherapy, or those with any serious medical conditions were excluded from this study.

### 2.2. Tomotherapy

All patients were instructed to breathe normally without control. Using a body net to fix the supine patients, an enhanced CT scan was used for location. Scanning range was from the mandible to 3 cm below the diaphragm, including the entire lung tissue. Slice thickness and slice gap were all 5 mm. Scanning image was sent directly onto the HT Hi-Art version 5.1.4 Accuray planning system (Accuray Company, Sunnyvale, California, U.S.A.) through the network with 4 s scanning speed for each level. Monaco version 5.11.01 software (Elekta, Stockholm, Sweden) was used to draw the target region and organs at risk (OARs). The pulmonary tumors were performed in the lung windows (window width: 1500–1700 HU; window center: -300 HU) and the mediastinal lymph nodes were delineated in the mediastinal windows (window width: 300 HU; window center: 60 HU). The gross target volume (GTV) was defined as the primary tumor and lymph nodes greater than 1.5 cm in the short axis in the enhanced CT or PET/CT; the clinical target volume (CTV) included extra 5 mm around the GTV; the planning target volume (PTV) included an extra 5 mm margin around the CTV. Image guidance was performed every time before treatment. We did not consider the impact of respiratory movements on inside target volume (ITV). Respiratory gating system was not used in the study. The prescription dose for GTV and CTV were 70 Gy and 60 Gy administered in 15–25 fractions over a period of 3–5 weeks. Patients with older age, severe multiple comorbidities, higher level of lymph node metastases, and large tumor volumes received more fractions treatment in order to reduce higher dose volume in the normal lung tissues and OARs.

The relative volume of the main bronchi, esophagus, and trachea receiving more than 55 Gy was required not to exceed 30%; the maximum dose to the spinal cord was not more than 45 Gy; the acceptable dose of the V20 for total lungs was ≤30%; the relative volume of heart receiving more than 50 Gy did not exceed 50%.

### 2.3. Follow-Up and Evaluation

Patients were assessed by physical examination and chest CT scan. The first follow-up was at 4–6 weeks after treatment; subsequent follow-ups were at 3^rd^, 6^th^, 9^th^, and 12^th^ in the first year, and every 6 months for the next two years. PET/CT was also recommended in all patients between 3–6 months in the first year or if tumor relapse was suspected. Local disease recurrence (LR) was defined as CT evidence of tumor progression in the same lobe or if the PET/CT images showed SUVmax >5. Regional disease recurrence (RR) was defined as occurrence of any intrapulmonary lymph node metastasis. Distant metastasis (DM) was defined as occurrence of any metastasis outside the lungs or any tumor seeding in a different lung lobe. The first date as the time of tumor progression was the time when the PET/CT or CT image demonstrated abnormality. More details are available in our previously published article [8]. Acute radiation pneumonitis (RP) was defined as pneumonitis occurring during the first 90 days of tomotherapy; late RP was defined as pneumonitis occurring more than 90 days after the start of radiotherapy. The Common Terminology Criteria for Adverse Events version 4.02 (CTCAE, U.S. Department of Health and Human Services, National Institutes of Health National Cancer Institute) and Radiation Therapy Oncology Group (RTOG) were used to classify the occurrence of acute and late PR, respectively.

### 2.4. Statistical Analyses

OS was measured from the first day of tomotherapy to the day of death or last follow-up. PFS, LR, RR, and DM were calculated from the first day of tomotherapy to the first day of the tumor progression or death. All statistical analyses were performed using the SPSS statistical software version 20.0 (IBM Corporation, Chicago, USA) or Graphpad Prism 8.3 software (LLC, California, USA). Pearson chi-square test and Fisher's exact test were used to compare the characteristics of patients. The Kaplan-Meier method was used to generate the OS and PFS curves; the optimal cutoff values of continuous variables were obtained by receiver operating characteristic (ROC) curve analysis. Univariate analyses and multivariate analyses were performed using the Cox proportional hazard model. Factors considered to influence the prognosis (such as history of chemotherapy and targeted drug therapy) or those associated with *p* values <0.2 in univariate analyses were included in the multivariate analyses. All significance tests were 2-tailed and *p* values <0.05 were considered indicative of statistical significance.

## 3. Results

The median age of patients was 69 years (range 50–86). Twenty-one patients had confirmed squamous cell carcinoma, 12 patients had confirmed adenocarcinoma, and the rest had other types. In 22 patients, the tumor was located in the central part, while 21 patients had peripheral tumors. Fifteen patients had undergone chemotherapy before tomotherapy, while 28 patients had not undergone chemotherapy (Table 1).

**Figure 1:**
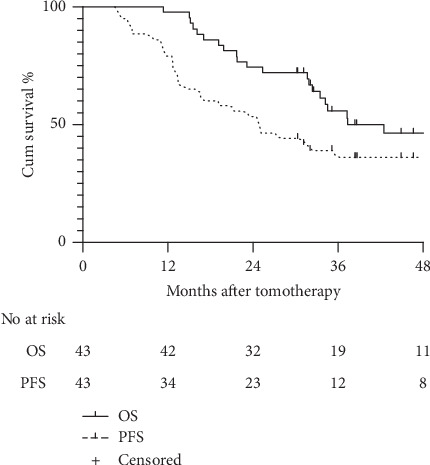
Curves illustrating the overall survival and progression-free survival in the cohort.

**Figure 2:**
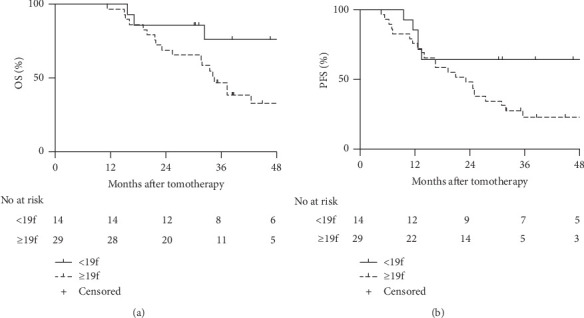
Overall survival (OS) rate and progression-free survival (PFS) rate in the two groups. (a) OS curves. (b) PFS curves.

The median OS time was 34.23 (range, 11.33–99.33) months, while the median PFS time was 25.00 (range, 4.6–99.33) months. The estimated 1-, 2-, and 3-year OS rates were 97.7%, 74.4%, and 55.9%, respectively, while the 1-, 2-, and 3-year PFS rates were 79.1%, 53.5%, and 36.1%, respectively (Figure 1). According to the ROC curve, the optimal cutoff value of the dose regimen was 19; therefore, we divided the study population into two groups (<19f vs. ≥19f). Fourteen patients received tomotherapy <19 fractions, while 29 patients received ≥19 fractions. There were no significant between-group differences with respect to gender, age, TNM stage, histology, location, chemotherapy history, targeted therapy, or KPS (Table 1). The estimated 1-, 2-, 3y- OS rates in the <19f group were 100%, 85.7%, and 76.2%, respectively; the corresponding rates in the ≥19f group were 96.6%, 69.0%, and 46.9%, respectively (Figure 2(a)). The PFS rates in <19f group were 85.7%, 64.3% and, 64.3%, while the corresponding rates in the ≥19f group were 75.9%, 48.3%, and 23.0%, respectively (Figure 2(b)).

Table 2 shows the results of univariate analysis and multivariate analysis (Cox proportional hazards model) to identify factors associated with OS, PFS, LR, RR, and DM. Both gender and dose regimen were associated with poorer OS (*p* < 0.05), while the dose regimen was the only factor associated with poorer PFS and DM (*p* < 0.05). Chemotherapy and targeted therapy were associated with poorer RR (*p* > 0.05) in univariate analysis; however, no significant association was observed in multivariate analysis. None of the factors showed an association with LR in univariate or multivariate analyses.

Table 3 shows the pattern of treatment failure and survival rate after tomotherapy. In 31 patients who showed disease progression, the original tumor recurrence was proven as LR in 2 patients (4.7%), RR in 5 patients (11.62%), and DM in 24 patients (55.81%). Two patients showed both recurrence and metastatic involvement of different organs after tomotherapy. One of the two patients had hepatic metastasis in a very short time, followed by recurrence at the original tumor site at 7.76 months; subsequently, RR and intracranial lymph gland metastases were detected at 14.93 months and 30 months, respectively. The patient died of multiple organ failure at 42.46 months after the initial tomotherapy. The other patient developed intrapulmonary metastases at 9.53 months after initial tomotherapy, followed soon by mediastinal lymph node metastasis; the patient died due to multiple metastases at 19.26 months. For the 24 patients who had an initial DM, the most common sites were pulmonary (37.5%) and multisystemic metastasis (41.67%); other sites were liver (4.17%), brain (8.33%), and bone (8.33%). To summarize, twenty-one patients (48.8%) died due to lung cancer, one patient died (2.3%) due to other disease, and two patients (4.7%) died due to unknown reasons.

Table 4 shows the rates of acute and late toxicity in all patients, such as hematological toxicity, esophagitis, dermatitis, and pneumonia. We focused on the incidence of RP rate and found that 7 patients experienced grade 1-2 treatment-related acute PR. Only 1 patient developed grade 2 acute PR in the <19f group, while 6 patients developed grade 1-2 acute PR in the ≥19f group. Only one of the patients experienced grade 3 acute hematological toxicity. None of the patients developed grade 4 or 5 toxicity during the acute phase. Five patients experienced grade 1-2 treatment-related late PR (2 patients in the <19f group and 3 patients in the ≥19f group). Three patients developed grade 3 late PR. None of the patients in either groups developed grade 4 or 5 late toxicity. We also compared the ratio of V5, V10, V20, V30, V40, and the average lung dose (MLD) of the left lung, the right lung, and the total lungs between patients with or without lung injury; we did not observe any significant difference between the two groups (*p* < 0.05, data not shown).

## 4. Discussion

In this study, hypofractionation with tomotherapy prolonged the overall survival time of patients with stage III NSCLC compared with traditional treatment. Moreover, this treatment reduced the occurrence of various complications during the perioperative period, thereby reducing the risk of death. Therefore, our findings suggest that hypofractionation with tomotherapy may be an effective alternative to surgery.

Currently, concurrent chemoradiotherapy is the standard treatment for patients with stage III NSCLC who cannot be operated [9]. However, the therapeutic outcomes for these patients are still poor. In the RTOG 0617 study, although the total dose was increased to 74 Gy, the single dose of 2 Gy had never changed, and the conventional radiotherapy mode was still used. The biological effective dose (BED) only increased from the original 72-79.2 Gy to 88.8 Gy. Since the body condition worsened after chemotherapy, the application of radiotherapy was restricted. Finally, the high-dose group (74 Gy) had to be terminated earlier with no evidence of improved outcomes [10]. Actually, small-scale studies have shown that the use of hypofractionated radiotherapy can significantly prolong the survival time of patients with locally advanced NSCLC without increasing the toxicity. In 2013, Oh et al. reported that chemotherapy concurrent with radiotherapy (2.4 Gy/f) can achieve a median survival time of 27.3 months [11]. In 2016, He et al. reported a median survival time of 31.6 months with the use of tomotherapy (60 Gy/20f) in patients with stage III NSCLC; the 1-year and 2-year survival rates were 88.2% and 58.1%, respectively [12]. In a study by Walraven et al., high-dose radiotherapy (66 Gy/24f) with cisplatin was found to prolong the median survival time to 31.5 months; the 1-year and 5-year survival rates were 74.5% and 37.3%, respectively [13]. All the above studies suggest that increasing single dose may help improve the survival time. The median single dose in our study was 3.5 Gy (2.8–4.67 Gy), which helped prolong the median survival time to 34.23 months; in addition, the 3-year OS rate (55.9%) was comparable or even better than the earlier results.

The modern radiotherapy technology has made it possible to deliver high-dose radiation to the target tissue while ensuring low dose to the surrounding normal tissues; this has made the SBRT as the standard treatment for patients with early-stage NSCLC [6–8]. In a retrospective multicenter clinical study in Japan, a minimum effective BED of 100–105 Gy was associated with a local recurrence rate of only 8.1%. However, under the opposite condition, the local recurrence rate is up to 26.4%. In our previous study, we used *γ*-SBRT technique with the mode of GTV/CTV/70 Gy/60 Gy/10 f (BED: 119 Gy) for patients with stage I/II NSCLC; the 3-year LC rate was 95% and the 5-year OS rate was 60.3% [8]. Nonetheless, it does not mean that the higher the BED, the better is the therapeutic outcome; excessive BED will greatly aggravate the damage to normal tissues with no concomitant increase in survival time. Corso et al. analyzed and compared the trend of dose prescriptions for 5246 patients with stage I NSCLC from 2004 to 2011 in America. They found that with the passage of time, the mode of 54–60 Gy/3 f was decreased with a concomitant increase in the mode of 50 Gy/5 f [14]. The probable reason for this trend is the increased adverse effects associated with higher BED. Therefore, the phenomenon of greater treatment benefit with higher BED is achievable only under certain conditions. In our study, the BED was between 89.6 Gy and 102.69 Gy, with the median BED reaching up to 94.5 Gy (close to 100 Gy); therefore, the results were promising.

The main cause of tumor progression is either poor local control or distant metastasis. Both study groups (<19f vs. ≥19f) showed excellent local control rates (100% vs. 93.10%, *p* = 0.322) and regional control rates (85.71% vs. 89.65%, *p* = 0.775). Only two patients developed local recurrence at 5.63 months and 7.76 months after treatment, while five patients developed regional recurrence within 3 years. We further found that the therapeutic effect in the <19 f group was better with higher BED (102.69 Gy) as compared to that in the ≥19 f group. Moreover, the dose regimen was the only independent prognostic factor for OS, PFS, and DM. This phenomenon is likely attributable to the activation of immunogenic system and the occurrence of “abscopal effect” after hypofractional radiation [15]. The exposure of tumor antigens increased due to constant production of new antigens. The high expression of immunoreactive proteins promotes the maturation and migration of antigen-presenting cells and causes the antitumor immune response of the body. All these factors contribute to cancer cell apoptosis and reduce distant metastasis. The other possible explanation for poor effects in ≥19 f group may be the lack of sequential chemotherapy as we did not persist with chemotherapy in these patients.

The occurrence of RP is the typical stumbling block to enhance target dosage and suppress tumor growth. Therefore, the long-term survival rate of lung cancer patients can be improved by increasing the target dose and minimizing RP. The reported incidence of RP in NSCLC patients treated with radical radiotherapy is 13–37%; however, in patients with stage III NSCLC, the incidence of RP may be higher because of concurrent chemoradiotherapy. In our study, the incidence of acute and late radiation lung injury was 16.27% and 18.60% (including 3 patients who had grade 3 injury). The incidence of late RP is similar to that reported by Yan et al. [16]. Yorke et al. showed that V5-V40 in both the total lung and the impaired lung are associated with radiation-induced pneumonia [17]. Shen et al. showed the composite factors combined with the lung V5, V20, and total MLD may directly influence the occurrence of RP; the rate of acute RP was significantly decreased when the V5 and V20 were less than 60% and 28%, and the total lung MLD was less than 14 Gy [18]. Jo et al. found that the critical value of grade 2-3 radiation pneumonia can be defined as V5 <65% [19]. We compared the dose of impaired lung and total lung between the groups with and without PR and found no significant between-group difference with respect to V5-V40 and MLD. We attribute the relatively low incidence of RP to the relatively low radiation doses of normal pulmonary; another important reason was that we did not use concurrent chemotherapy in our study, because Yan et al. reported that chemotherapy was a significant factor associated with severe acute RP [16].

Some limitations of our study should be improved. In several studies, tumor volume, or even volume changes during treatment were associated with the overall survival of patients [20–22]. Owing to the limited number of patients, we did not perform subgroup analysis nor did we explore the influence of subtle factors that may affect the prognosis of patients with stage III NSCLC. Secondly, some studies have shown that radiotherapy cannot completely eliminate tumor immune tolerance; therefore, the therapeutic effect was limited. Radiotherapy administered in combination with immunotherapy (ipilimumab, PD-1/PD-L1, GM-CSF, etc.) can be more effective in killing the tumor cells [23–25]. Golden et al. reported four patients with lung cancer who were treated with radiotherapy (35 Gy/10 f in target area) in combination with granulocyte macrophage colony-stimulating factor; the other tumor decreased by more than 30% outside of target [25]. Therefore, an appropriate radiotherapy dose regimen with optimal timing of immunotherapy may maximize the therapeutic effect in patients with NSCLC; this approach will be investigated in our future study. Lastly, there is a paucity of prognostically-relevant clinical indicators; in particular, there is no good predictive index to evaluate the radiation injury of OARs. Application of radiomics technology may provide powerful indices for predicting outcomes (survival, recurrence, radiation injury, etc.); it can also provide more objective predictors of radiation-induced lung injury following hypofractionational radiation for stage III NSCLC [26].

## 5. Conclusions

Our findings suggest that hypofractionation with tomotherapy is an effective treatment option for patients with stage III NSCLC who were medically inoperable or refuse concurrent chemotherapy; these patients may also benefit from the reduced incidence of toxicity.

## Figures and Tables

**Table 1:** Clinical characteristics and outcomes of the 43 enrolled patients.

Characteristic	No. of patients	Dose regimen		*P*
		<19 f	≥19 f	
TNM stage				
IIIa	27	9	18	0.888
IIIb	16	5	11	
T stage				
T1-2	11	3	8	1.000
T3-4	32	11	21	
N stage				
N0-2	33	11	22	1.000
N3	10	3	7	
Age				
<80.5	37	13	24	0.645
≥80.5	6	1	5	
Location				
Central	22	7	15	0.916
Peripheral	21	7	14	
Chemotherapy history				
Yes	15	5	10	1.000
No	28	9	19	
Gender				
Male	31	10	21	1.000
Female	12	4	8	
Targeted drug				
Yes	6	2	4	1.000
No	37	12	25	
KPS				
<90	27	7	20	0.228
≥90	16	7	9	
Histology				
Adenocarchnoma	12	2	10	0.382
Squamous cell	21	8	13	
Others	10	4	6	

Data presented as frequencies unless indicated otherwise. KPS: Karnofsky performance score.

**Table 2:** Univariate and multivariate Cox proportional hazards regression analysis for OS and PFS.

Item	OS	PFS	LRFS	RRFS	DMFS
	*P*	*P*	*P*	*P*	*P*
Univariate analysis					
Age (<80.5 y vs. ≥80.5 y)	0.235	0.743	0.566	0.363	0.876
Gender (male vs. female)	0.030	0.202	0.375	0.232	0.126
KPS (<87.5 vs. ≥87.5)	0.985	0.798	0.272	0.938	0.853
Histology (adenocarchnoma vs. squamous cell vs. others)	0.470	0.528	0.342	0.564	0.941
Dose regimen (<19 f vs. ≥19 f)	0.022	0.025	0.322	0.775	0.015
Location (central vs. peripheral)	0.246	0.401	0.162	0.492	0.438
Chemotherapy (yes vs. no)	0.302	0.172	0.664	0.016	0.060
TNM stage (IIIa vs. IIIb)	0.953	0.428	0.272	0.979	0.806
T stage (T1-2 vs. T3-4)	0.676	0.725	0.436	0.478	0.992
N stage (N0-2 vs. N3)	0.845	0.436	0.433	0.832	0.661
Molecular targeted therapy	0.865	0.271	0.145	0.042	0.699
Multivariate analysis (Cox)					
Gender (male vs. female)	0.033	0.155	0.984	0.714	0.108
Molecular targeted therapy	0.882	0.075	0.124	0.143	0.139
Chemotherapy (yes vs. no)	0.706	0.191	0.862	0.357	0.099
Dose fraction (<19 f vs. ≥19 f)	0.020	0.022	0.985	0.667	0.014

OS: overall survival; PFS: progression-free survival; LRFS: local recurrence-free survival; RRFS: regional recurrence-free survival; DMFS: distant metastasis-free survival.

**Table 3:** Patterns of treatment failure and survival rate after tomotherapy.

Event	Actual incidence %	Estimated cumulative incidence %		
		1 year	2 year	3 year
Overall survival		97.7	74.4	55.9
Progression-free survival		79.1	53.5	36.1
Local disease recurrence	4.7	4.7 (2)	4.7	4.7
Regional disease recurrence	11.62	0.0	10.5 (4)	15.7 (5)
Distant disease recurrence	55.81	18.7 (8)	40.2 (17)	57.9 (24)
Any progression	72.09	20.9	46.5	63.9

**Table 4:** Adverse effects after tomotherapy.

Adverse effects	Grade 1-2 (%)	Grade 3 (%)
Acute		
Hematological toxicity	9 (20.93)	1 (2.32)
Fatigue	11 (25.58)	0
Esophagitis	8 (18.60)	0
Dermatitis	1 (2.32)	0
Pneumonia	7 (16.27)	0
<19 f	1 (2.32)	0
≥19 f	6 (13.95)	0
Late		
Hematological toxicity	5 (11.63)	0
Fatigue	4 (9.30)	0
Esophagitis	1 (2.32)	0
Pneumonia	5 (11.63)	3 (6.98)
<19 f	2 (4.65)	0
≥19 f	3 (6.98)	3 (6.98)

## Data Availability

The data used to support the findings of this study are available from the corresponding author upon request.
